# Non-specific symptoms and post-treatment Lyme disease syndrome in patients with Lyme borreliosis: a prospective cohort study in Belgium (2016–2020)

**DOI:** 10.1186/s12879-022-07686-8

**Published:** 2022-09-28

**Authors:** Laurence Geebelen, Tinne Lernout, Brecht Devleesschauwer, Benoît Kabamba-Mukadi, Veroniek Saegeman, Leïla Belkhir, Paul De Munter, Bénédicte Dubois, Rene Westhovens, Humtick Hospital Group, Jean-Baptiste Giot, Philippe Léonard, Riet Vangheluwe, Grégoire Wieërs, Jean-Christophe Marot, Frédéric Evrard, Bénédicte Delaere, Séverine Noirhomme, Els Binnemans, Johan Vanhoof, Herman Van Oyen, Niko Speybroeck, Katrien Tersago

**Affiliations:** 1 Department of Epidemiology and Public Health, Sciensano, Brussels, Belgium; 2 Institute of Health and Society (IRSS), Université Catholique de Louvain, Woluwe-Saint-Lambert, Belgium; 3 Department of Translational Physiology, Infectiology and Public Health, Ghent University, Merelbeke, Belgium; 4 Laboratory of Medical Microbiology, Université Catholique de Louvain, Brussels, Belgium; 5 Department of Microbiology, AZ Nikolaas, Sint-Niklaas, Belgium; 6 Cliniques Universitaires Saint-Luc Université Catholique de Louvain, Brussels, Belgium; 7 Department of General Internal Medicine, University Hospitals Leuven, Leuven, Belgium; 8 Department of Microbiology, Immunology and Transplantation, KU Leuven, Leuven, Belgium; 9 Department of Neurology, University Hospitals Leuven, Leuven, Belgium; 10 Department of Rheumatology, University Hospitals Leuven, Leuven, Belgium; 11 Department of Development and Regeneration, Skeletal Biology and Engineering Research Centre, KU Leuven, Leuven, Belgium; 12 Department of Public Health and Primary Care, Ghent University, Ghent, Belgium; 13 Flemish Agency for Care and Health, Brussels, Belgium

**Keywords:** Lyme borreliosis, Erythema migrans, Disseminated Lyme borreliosis, Post-treatment Lyme disease syndrome, Persisting non-specific symptoms

## Abstract

**Background:**

Patients with Lyme borreliosis (LB) may report persisting non-specific symptoms such as fatigue, widespread musculoskeletal pain or cognitive difficulties. When present for more than 6 months and causing a reduction in daily activities, this is often referred to as post-treatment Lyme disease syndrome (PTLDS). This study aimed to compare the occurrence of symptoms between LB patients and controls, to estimate the proportion of LB patients developing PTLDS and to identify risk factors.

**Methods:**

A prospective cohort study was set up including three subpopulations: patients with an erythema migrans (EM) (i) or disseminated/late LB (ii) and a non-LB control group (iii). At 6- and 12-months follow-up, the occurrence of several symptoms, including six symptoms used to define PTLDS, i.e. muscle pain, joint pain, fatigue, memory problems, difficulties concentrating and problems finding words, and impact on daily activities, was compared between LB patients and controls. Finally, the proportion of LB patients developing PTLDS as defined by the Infectious Disease Society of America was estimated, including a time frame for symptoms to be present.

**Results:**

Although the risk of presenting PTLDS-related symptoms was significantly higher in EM patients (n = 120) compared to controls (n = 128) at 6 months follow-up, the risk of presenting at least one of these symptoms combined with impact on daily activities was not significantly higher in EM patients, at either 6- or 12-months follow-up. A significant association was found between disseminated/late LB (n = 15) and the occurrence of any PTLDS-symptom with an impact on daily activities at both time points. The proportion of patients with PTLDS was estimated at 5.9% (95% CI 2.7–12.9) in EM patients and 20.9% (95% CI 6.8–64.4) in patients with disseminated/late LB (RR = 3.53, 95% CI 0.98–12.68, p = 0.053). No significant risk factors were identified, which may be explained by small sample sizes.

**Conclusions:**

In our study, PTLDS was present in both LB cohorts, yet with a higher percentage in disseminated/late LB patients. Additional research is needed into risk factors for and causes of this syndrome. In addition, development and validation of standardized methods to assess the PTLDS case definition, easily applicable in practice, is of great importance.

**Supplementary Information:**

The online version contains supplementary material available at 10.1186/s12879-022-07686-8.

## Introduction

Lyme borreliosis (LB) is an important tick-borne disease caused by spirochetes of the *Borrelia burgdorferi* sensu lato (s.l.) complex. As the spirochetes can affect different sites of the body, it can manifest with a broad variety of clinical symptoms, yet asymptomatic infections also occur [1, 2]. The most common manifestation is an erythema migrans (EM), an early-localized infection, possibly accompanied with flu-like symptoms. When left untreated, early disseminated LB or late LB may develop, amongst others, as multiple erythema migrans (MEM), Lyme neuroborreliosis (LNB), Lyme arthritis (LA), Lyme carditis or acrodermatitis chronica atrophicans (ACA) [1–3]. In addition to these objectively identifiable manifestations, it has been reported that a subset of patients continued to experience non-specific symptoms, even despite adequate antibiotic treatment. Frequencies of these symptoms reported in literature vary widely, between 5 and 36% in EM patients, with the highest frequency in a study on EM patients also presenting with systemic symptoms (e.g. viral-like symptoms) [4–11], and up to about 50% in some studies in LNB patients [12–15]. However, substantial heterogeneity exists in the case definitions used, the study designs and the population under study. In order to better delineate this group of patients and to improve research on the occurrence and pathogenesis of these symptoms, a case definition for post-Lyme disease syndrome (PLDS), now mostly referred to as post-treatment Lyme disease syndrome (PTLDS), was proposed by the Infectious Disease Society of America (IDSA) in 2006 [16]. This definition includes the onset of fatigue, widespread musculoskeletal pain or complaints of cognitive difficulties within 6 months of a proven *B. burgdorferi* infection, symptoms persisting for ≥ 6 months (continuous or relapsing) after appropriate antibiotic therapy which led to resolution or stabilization of objective manifestations, and the symptoms being of such severity that, when present, they result in substantial reduction in previous levels of occupational, educational, social, or personal activities. In addition, several exclusion criteria were proposed [16]. As the symptoms of PTLDS are also very prevalent in the general population, controversy still exists on the frequency, cause and treatment of the syndrome. For the latter, several randomized controlled treatment trials showed insufficient evidence of the benefit of prolonged antibiotic treatment in patients with persisting symptoms [17–21].

The aim of this study was twofold: first, to compare non-specific symptoms as such and symptoms impacting daily activities between LB patients and a non-LB control group at 6 and 12 months follow-up; second, to estimate the proportion of PTLDS in two groups of LB patients–patients with an erythema migrans (i) and disseminated or late LB (ii)—according to the full IDSA case definition including the time frame proposed, and to identify possible risk factors. To allow comparison with previously reported results, different steps were followed to present and discuss the study results.

## Methods

### Study design and participant selection

This study was part of a larger project, the HUMTICK study, for which the initial study protocol has been described previously [22]. HUMTICK is a prospective cohort study in which patients diagnosed with an EM (i) or disseminated/late LB manifestations (ii) were followed up for 6 to 24 months after diagnosis and treatment, and a non-LB control group (iii) was concurrently followed up during 6 to 12 months. Participants, aged 18 years or older, were included between June 2016 and December 2019. EM patients were recruited by a network of 200 general practitioners and patients with disseminated/late LB manifestations were recruited by medical specialists in 8 hospitals, all located in areas in Belgium that are highly endemic for tick bites and LB. Case definitions for inclusion were published in the study protocol [22] and can be found in Additional file 1 [23, 24]. In these, EM is clinically diagnosed by the GP whereas other manifestations are based on both clinical and laboratory criteria. Multiple EM, although a disseminated manifestation, was included in the group of EM patients (i). If the EM diameter was < 5 cm and no tick bite was recalled or the delay in EM appearance was less than 2 days (if known), the diagnosis was classified as possible EM and the patient was not included in the baseline analysis. LNB patients included as a case by the hospital based on high suspicion, but without a cerebrospinal fluid analysis performed at diagnosis were similarly not included in the baseline analysis. Participants of the non-LB control group were selected by patients among persons in their environment with the same gender and age ± 5 years and no prior LB diagnosis. As not all patients were able to provide a control person, additional controls were recruited by the participating controls (with the same requirements) and through online invitations of persons in gender and age categories lacking controls at the end. Pregnant patients were excluded.

### Evaluation of participants

LB patients were asked to fill in a questionnaire at diagnosis (T0), 1 and 3 months later (T1 and T3), and at 6, possibly 12 and 24 months after treatment completion (T6, T12 and T24), depending on the moment of enrollment and time left until the end of the study period. In line with the IDSA case definition for PTLDS, the questionnaires assessed the presence of subjective symptoms, as well as the impact on occupational, educational, social or personal activities, hereafter referred to as daily activities [16]. Patients’ health before and during the onset of their LB was assessed in the first questionnaire and post-treatment health was assessed in the follow-up questionnaires from 3 months onwards. The same questions were answered by the control group at T0 (inclusion) and T6, possibly at T12 (depending on the moment of enrollment).

#### Symptoms

Six symptoms were used throughout the study to assess the symptoms included in the IDSA definition for PTLDS, henceforth referred to as PTLDS-related symptoms: muscle pain (> 1 part of the body), joint pain (> 1 part of the body), fatigue, memory problems (forgetfulness), difficulties concentrating and problems finding words. At follow-up, five additional non-specific symptoms—headache, sensory disorders (e.g. tingling), night sweats (with waking up), excessive sleep and troubles falling asleep—were added to the list, as these have been suggested in literature to be related to PTLDS [25]. Presence of swollen joints was added as an objective symptom, leading to a 12-item symptom list. For each symptom, patients reported whether or not it had regularly been present (pre-Lyme or since the previous questionnaire), the cause if known, and for each symptom present, if it was more severe, equally severe or less severe at follow-up compared to their pre-Lyme general health (or pre-participation health for the control group). Furthermore, three standardized questionnaires, assessing the severity of the PTLDS-related symptoms were added:

1. SF-36 bodily pain (SF-36 BP) subscale to assess widespread musculoskeletal pain during the past 4 weeks [26, 27].
2. SF-36 vitality (SF-36 VT) subscale to assess fatigue during the past 4 weeks [26, 27].
3. Cognitive Failure Questionnaire (CFQ) to assess cognitive difficulties during the past months [28].

To assess pre-Lyme health (at T0), the periods covered by the questionnaires were adapted to 4 weeks (SF-36) or the months (CFQ) before the tick bite or before the start of LB complaints.

#### Impact on daily activities

At follow-up, a modified version of the global activity limitation indicator (GALI, a three-level multiple choice question) was used to assess the impact of the 12 symptoms on the patient’s life, i.e., patients were asked whether they were limited in their daily activities (e.g. work, school, hobbies, or others) due to these symptom(s) [29, 30]. Furthermore, a question on having problems with usual activities in general (e.g. work, study, housework, family or leisure activities) was added, assessing the past 4 weeks. This question is part of the standardized EQ-5D-5L questionnaire [31, 32].

### Outcome definitions

#### Comparison of patients vs. controls at follow-up

Following a step-wise approach, different outcomes were defined to compare between LB patients and the non-LB control group at 6- and 12-months follow-up. Firstly, the new occurrence or worsening of each symptom of the 12-item symptom list was compared as such. Secondly, the comparison of symptoms was narrowed down to the occurrence of at least one of the six PTLDS-related symptoms and an impact on daily activities was added as a criterion. Details on the exact definitions used are shown in Table 1.

**Table 1:** Outcome definitions in the comparison of patients vs. controls at 6 and 12 months follow-up, HUMTICK study, Belgium, 2016–2020

1. **Occurrence of new or worsened non-specific symptoms (12 symptoms):**
**New or worsened symptom**^**a**^** =**
• Regularly present since the previous questionnaire AND • “More severe” compared to pre-Lyme or pre-participation health AND • Worse standardized questionnaire score for symptom present (only for the 6 PTLDS-related symptoms based on SF-36 BP, SF-36 VT or CFQ)
**2. Occurrence of any of the 6 PTLDS-related symptoms:**
**At least one of the 6 PTLDS-related symptoms new or worsened**
• Defined as above^a^
**+ Impact daily activities =**
• Limited or strongly limited on the modified GALI AND • At least slight problems with daily activities on the question of the EQ-5D-5L (= level 2–5)

^a^Note that these symptoms can have other possible causes as no exclusion was performed at this stage

#### PTLDS in LB patients

**Fig. 1:**
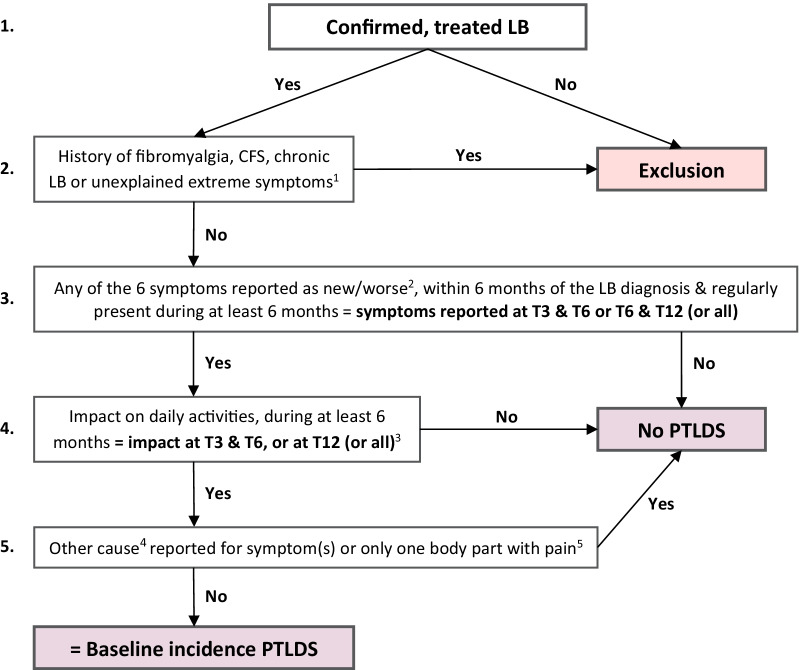
Decision tree for defining post-treatment Lyme disease cases following IDSA definition [16]. LB: Lyme borreliosis; CFS: chronic fatigue syndrome; T3: 3 months after diagnosis; T6 and T12: 6 and 12 months after treatment. ^1^Unexplained or undiagnosed extreme fatigue, widespread musculoskeletal pain or cognitive difficulties. ^2^Widespread musculoskeletal pain expressed as muscle pain (1) or joint pain (2) at more than one place of the body, fatigue (3), or cognitive difficulties expressed as memory problems (4), difficulties concentrating (5) or problems finding words (6). ^3^The impact on daily activities had to be present during at least 6 months, but not necessarily start within 6 months. ^4^Causes explaining the new occurrence or worsening of a symptom reported by the patient as cause for the symptom (e.g. acute diseases, new comorbidities, flare-up of comorbidity already present,…). ^5^Based on an open question asking to report body parts with pain

As a third step, to estimate the proportion of patients developing PTLDS after an EM (i) and after disseminated/late LB manifestations (ii), additional criteria of the IDSA case definition were applied to the two cohorts as shown in Fig. 1. In order to include the time frame (symptom start < 6 months, duration ≥ 6 months), different time points were combined in the analysis. In the baseline scenario, the definitions as described in Table 1 were used to define new or worsened symptoms and impact on daily activities. In addition to the baseline scenario, the incidence of PTLDS was estimated following four alternative scenarios using different inclusion or outcome criteria (see further).

The Additional file 2 gives a detailed description of all the criteria of the IDSA definition for PTLDS and the (practical) adaptations made for the HUMTICK study.

### Statistical analysis

All analyses were performed in R version 3.6.3 [33]. Due to the smaller sample size than initially foreseen and as patients and controls were not individually matched, changes were made to the described data analysis in the study protocol [22]. Patient demographics were compared with controls using two-sided Pearson’s Chi-squared tests, Fisher’s exact tests when appropriate (expected frequency < 5) or t-test for continuous variables.

#### Comparison of patients vs. controls at follow-up

Both for the comparison of the occurrence of each symptom separately, as for the occurrence of at least one PTLDS-related symptom (with impact on daily activities), risk ratios and 95% confidence intervals (CI) were calculated by univariate log-binomial regression analysis. Multivariable analysis could not be performed, as the number of events was too small. Mean standardized questionnaire scores of the LB patient groups were compared to those of controls using linear regression analysis. For the comparisons at 12 months, only participants with follow-up were included in the analysis. Other missing data were accounted for using multivariate imputation by chained equations (20 repetitions) using the MICE package in R [34]. All regression analyses were performed on each of the imputed databases and results were pooled using Rubin’s rules which calculate mean results from all imputed databases, complemented with confidence intervals and p-values reflecting the within- and between-imputation variance [34]. P-values < 0.05 were considered significant.

#### PTLDS in LB patients

The binary outcome PTLDS combines different time points following the IDSA definition. Since this definition includes symptom start within 6 months after diagnosis, PTLDS status was known for all patients without any of the six symptoms at T6, even without follow-up at T12. This was also the case for patients that fulfilled the complete PTLDS definition (i.e. including duration 6 months and impact) at T6. PTLDS status could however not be evaluated for patients with symptoms at T6 who did not (yet) fulfill the complete PTLDS definition and had no follow-up at T12 (n EM = 3; n DISS = 2), as these were still at risk of developing PTLDS. Hence, these patients were censored and the missing PTLDS-status was imputed by multiple imputation, instead of excluding them from the analysis (which would lead to bias). Log-binomial regression analysis was used to estimate the proportion of LB patients developing PTLDS, again performed on each imputed database and pooled using Rubin’s rules. Disseminated/late LB was analyzed as a risk factor for the development of PTLDS in a log-binomial univariate analysis including all LB patients (EM and disseminated/late LB). Furthermore, within the group of EM patients, associations of other potential risk factors with PTLDS were analyzed i.e. socio-demographic variables, comorbid illness and symptom presence before LB, symptom presence at diagnosis and the treatment prescribed. After univariate log-binomial regression analysis, variables with a p-value < 0.25 were included in a multivariable model on which backward exclusion was performed to retain only significant risk factors (multivariate Wald test, p-values < 0.05). Due to the small sample size and related computational problems, risk factors were not analyzed in the group of disseminated and late LB patients.

## Results

### Participant characteristics

Informed consent was received from 141 patients with EM, 29 with disseminated/late LB and 157 controls recruited for the study. After exclusion of patients without LB and controls with previous LB, participants with incomplete data at inclusion (i.e. part of questionnaire missing) and participants without follow-up until at least T6 (LTFU), 120 patients with an EM, 15 patients with disseminated/late LB and 128 control persons were included in the baseline analysis of the study. Characteristics of these participants are shown in Table 2. A flowchart for participant exclusion is given in Additional file 3. At T12, 94 EM patients, 11 disseminated/late LB patients and 81 controls had follow-up data. As only patients included at the beginning of this study could participate in the follow-up at T24, the number of participants with a follow-up of 24 months was low (25 EM patients and 6 disseminated/late LB patients). Hence, these data were not used for extensive analysis.

**Table 2:** Characteristics of participants included in the HUMTICK study, Belgium, 2016–2020

Category	EM patients N (%)	DISS patients N (%)	Controls N (%)	p-value EM vs. controls	p-value DISS vs. controls
Total	120	15	128	–	–
Gender				0.696	**< 0.001**
Male	46 (38.3)	13 (86.7)	46 (35.9)		
Female	74 (61.7)	2 (13.3)	82 (64.1)		
Age				0.723	0.328
18–29	10 (8.3)	1 (6.7)	14 (10.9)		
30–39	15 (12.5)	0 (0)	15 (11.7)		
40–49	17 (14.2)	4 (26.7)	20 (15.6)		
50–59	39 (32.5)	2 (13.3)	32 (25)		
60–69	23 (19.2)	7 (46.7)	32 (25)		
70+	16 (13.3)	1 (6.7)	15 (11.7)		
Region				0.520	0.329
Flanders	82 (68.3)	7 (46.7)	81 (63.3)		
Wallonia	34 (28.3)	8 (53.3)	40 (31.2)		
Brussels	2 (1.7)	0 (0)	6 (4.7)		
Not Belgium^a^	2 (1.7)	0 (0)	1 (0.8)		
Highest completed education				**0.017**	0.760
Lower	49 (40.8)	3 (20)	34 (26.6)		
Higher	71 (59.2)	12 (80)	94 (73.4)		
Work				0.414	0.696
Full-time	45 (38.1)	6 (40.0)	59 (46.5)		
Part-time	26 (22)	2 (13.3)	23 (18.1)		
Student	6 (5.1)	0 (0)	5 (3.9)		
Retired	32 (27.1)	6 (40)	36 (28.3)		
Not working, other	9 (7.6)	1 (6.7)	4 (3.1)		
Inclusion period				**0.005**	0.889
Spring/Summer	97 (80.8)	10 (66.7)	83 (64.8)		
Autumn/Winter	23 (19.2)	5 (33.3)	45 (35.2)		
Comorbidity					
Musculoskeletal disease	18 (15.1)	2 (13.3)	10 (7.8)	0.070	0.616
Heart disease	9 (7.6)	0 (0)	4 (3.1)	0.155	1.000
Pulmonary disease	8 (6.7)	1 (6.7)	3 (2.3)	0.126	0.361
Thyroid disorder	7 (5.9)	0 (0)	5 (3.9)	0.470	1.000
Other possibly impacting^b^	10 (8.4)	2 (13.3)	4 (3.1)	0.099	0.121
Other non-impacting^c^	30 (25.2)	3 (20)	26 (20.3)	0.358	1.000
Fibromyalgia, chronic fatigue syndrome	2 (1.7)	0 (0)	1 (0.8)	0.610	1.000
PTLDS-related symptoms in the months before LB or before participation					
Muscle pain	32 (27.1)	1 (6.7)	30 (23.4)	0.506	0.192
Joint pain	37 (31.1)	3 (21.4)	37 (28.9)	0.708	0.757
Fatigue	37 (30.8)	4 (26.7)	44 (34.9)	0.495	0.774
Memory difficulties	14 (11.9)	0 (0)	22 (17.3)	0.228	0.127
Concentration difficulties	15 (12.6)	0 (0)	13 (10.2)	0.559	0.363
Wording difficulties	19 (16.2)	0 (0)	21 (16.5)	0.950	0.363
Any of 6 above	61 (50.8)	6 (40.0)	86 (67.2)	**0.009**	**0.038**
N symptoms^d^	2.5	1.3	1.9	**0.011**	**0.035**

*EM* erythema migrans, *DISS* disseminated/late Lyme borreliosis, *N* number, *PTLDS* post-treatment Lyme disease syndrome, *LB* Lyme borreliosis

^a^Close to the Belgian border, EM diagnosis by GP in Belgium

^b^Other diagnosis with impact expected on fatigue, widespread pain or cognitive difficulties: sleep apnea, anemia, cancer within past 2 years, hemochromatosis, Ehler-Danlos, depression, Attention Deficit Disorder, hepatitis, Crohn’s disease, chronic pelvic pain

^c^Other diagnosis with no or limited impact expected on fatigue, widespread musculoskeletal pain or cognitive difficulties, amongst others gastro-esophageal or intestinal diseases, urinary, eye, skin, liver or gynaecological diseases

^d^Mean number of symptoms above, reported by participants with at least one of six symptoms

Compared to the control group, a significantly higher proportion of EM patients had a lower education (p = 0.017) and were included in spring/summer (p = 0.005). The disseminated/late LB group was heavily skewed towards men, hence the proportion of the latter was significantly higher compared to the control group (p < 0.001). None of the other demographics differed significantly between the groups but sample size for disseminated/late LB was very small. While no significant differences were found in the presence of the six PTLDS-related symptoms separately between patients (before LB) and controls (before participation), the proportion reporting any of the six symptoms was higher in control persons compared to LB patients (p = 0.009 and p = 0.038), whereas the mean number of symptoms, in those with at least one symptom, was higher in EM patients (p = 0.011) but lower in disseminated/late LB patients (p = 0.035).

Table 3 shows the manifestations of the LB patients at inclusion. Out of the 120 EM patients, 63.3% could remember a tick bite, with a median of 15 days between the bite and the diagnosis (range 1–102). The median time since first notice of EM equaled 7 days (range 0–90). For the disseminated/late LB group, 7 patients remembered a tick bite but only 4 remembered when it occurred. The median time between start symptoms and diagnosis equaled 32 days (range 4–165).

**Table 3:** Lyme borreliosis manifestations at inclusion, HUMTICK study, Belgium, 2016–2020

	EM patients (i)	Disseminated/late LB (ii)^c^
**Baseline**	**120**	**15**
EM	115	–^a^
MEM	5	–
LNB early	–	8
LA	–	2
Carditis	–	4^b^
ACA		1
**+ Possible (alternative scenario)**	**5**	**3**
EM	5	–
LNB^c^ early	–	2
LNB^c^ late	–	1

*EM* erythema migrans, *MEM* multiple erythema migrans, *LNB* Lyme neuroborreliosis, *LA* Lyme arthritis, *ACA* acrodermatitis chronica atrophicans, *LB* Lyme borreliosis

^a^Some patients reported an EM yet no clinical information was available as it was often no longer present at inclusion

^b^LA was reported in one carditis patient and unconfirmed LNB (without lumbar puncture) was reported in two out of four carditis patients (including the patient with carditis and LA)

^c^No lumbar puncture performed (n = 2) or only after treatment and negative (n = 1), but patient included by specialist based on clinical symptoms and other laboratory results

### Comparison of patients vs. controls at follow-up

#### Occurrence of new or worsened non-specific symptoms

Risk ratios for each symptom separately (new or worsened, regularly present since the previous questionnaire) in the patient groups compared to the control group at 6 and 12 months follow-up, are shown in Table 4.

**Table 4:** New or worsened symptoms in patients with Lyme borreliosis compared to controls at 6 and 12 months follow-up, HUMTICK study, Belgium, 2016–2020

Time point	EM N (%)	DISS N (%)	Controls N (%)	EM vs. controls		DISS vs. controls	
				RR (95% CI)	P-value	RR (95% CI)	P-value
Total N							
6 months	120	15	128				
12 months	94	11	81				
**PTLDS-related symptoms**^a^							
Muscle pain							
6 months	11 (9.2)	2 (13.3)	3 (2.3)	3.91 (1.11–13.77)	**0.034**	5.69 (1.02–31.84)	**0.048**
12 months	7.4 (7.9)	0 (0)	1 (1.2)	6.36 (0.79–51.27)	0.082	0 (0–Inf)	0.997
Joint pain							
6 months	7 (5.8)	5 (33.3)	4 (3.1)	1.87 (0.56–6.25)	0.310	10.67 (3.18–35.83)	**< 0.001**
12 months	11.6 (12.3)	4 (36.4)	6 (7.4)	1.66 (0.64–4.29)	0.296	4.91 (1.61–14.93)	**0.006**
Fatigue							
6 months	15.2 (12.7)	5 (33.3)	4 (3.2)	4.02 (1.36–11.86)	**0.012**	10.55 (3.14–35.44)	**< 0.001**
12 months	8 (8.5)	5 (45.5)	3 (3.7)	2.30 (0.62–8.45)	0.209	12.27 (3.33–45.17)	**< 0.001**
Memory difficulties							
6 months	6 (5.0)	0 (0)	3 (2.3)	2.13 (0.54–8.40)	0.277	0 (0–Inf)	0.995
12 months	6 (6.4)	0 (0)	2 (2.5)	2.59 (0.53–12.60)	0.238	0 (0–Inf)	0.996
Concentration difficulties							
6 months	4 (3.3)	0 (0)	1 (0.8)	4.27 (0.48–38.05)	0.193	0 (0–Inf)	0.997
12 months	5 (5.3)	1 (9.1)	0 (0.0)	Inf (0–Inf)	0.992	Inf (0–Inf)	0.997
Wording difficulties							
6 months	2 (1.7)	0 (0)	4 (3.1)	0.53 (0.10–2.88)	0.464	0 (0–Inf)	0.995
12 months	4 (4.3)	1 (9.1)	1 (1.2)	3.45 (0.39–30.69)	0.265	7.36 (0.48–113.68)	0.151
**Other symptoms**^b^							
Headache							
6 months	5 (4.2)	0 (0)	2 (1.6)	2.67 (0.52–13.59)	0.237	0 (0–Inf)	0.995
12 months	3 (3.2)	0 (0)	2 (2.5)	1.29 (0.22–7.64)	0.776	0 (0–Inf)	0.996
Sensory disorders							
6 months	7 (5.8)	1 (6.7)	1 (0.8)	7.47 (0.92–60.37)	0.059	8.53 (0.55–132.63)	0.125
12 months	3 (3.2)	0 (0)	0 (0.0)	Inf (0–Inf)	0.995	1.00 (0–Inf)	> 0.999
Night sweats							
6 months	3 (2.5)	0 (0)	3 (2.3)	1.07 (0.22–5.22)	0.936	0 (0–Inf)	0.995
12 months	2 (2.1)	0 (0)	6 (7.4)	0.29 (0.06–1.40)	0.122	0 (0–Inf)	0.993
Excessive sleeping							
6 months	5 (4.2)	0 (0)	3 (2.3)	1.78 (0.43–7.33)	0.424	0 (0–Inf)	0.995
12 months	3 (3.2)	0 (0)	2 (2.5)	1.29 (0.22–7.64)	0.776	0 (0–Inf)	0.996
Difficulties falling asleep							
6 months	2 (1.7)	1 (6.7)	5 (3.9)	0.43 (0.08–2.21)	0.312	1.73 (0.21–14.13)	0.609
12 months	4.8 (5.0)	0 (0)	3 (3.7)	1.36 (0.32–5.74)	0.675	0 (0–Inf)	0.996
Swollen joints							
6 months	2.3 (1.9)	1 (6.7)	2 (1.6)	1.20 (0.17–8.40)	0.850	4.27 (0.40–45.22)	0.226
12 months	2 (2.1)	0 (0)	3 (3.7)	0.57 (0.10–3.40)	0.539	0 (0–Inf)	0.996

*EM* erythema migrans, *DISS* disseminated/late Lyme borreliosis, *N* number, *RR* risk ratio, *CI* confidence interval, *PTLDS* post-treatment Lyme disease syndrome

^a^Worsened standardized questionnaire score for symptoms included in definition

^b^No standardized questionnaire score included in definition

At 6 months follow-up, new or worsened fatigue and muscle pain were significantly associated with previous LB with a risk ratio of 4.02 (95% CI 1.36–11.86, p = 0.012) and 3.91 (95% CI 1.11–13.77, p = 0.034) respectively for EM patients and a risk ratio of 10.55 (95% CI 3.14–35.44, p < 0.001) and 5.69 (95% CI 1.02–31.84, p = 0.048) respectively for disseminated/late LB patients. In the latter, at T6, the risk of joint pain was also significantly higher compared to controls with a RR of 10.67 (95% CI 3.18–35.83, p < 0.001). At 12 months follow-up, no symptoms were significantly higher in EM patients but fatigue and joint pain remained significantly associated with disseminated/late LB with a risk ratio of 12.27 (95% CI 3.33–45.17, p < 0.001) and 4.91 (95% CI 1.61–14.93, p = 0.006) respectively.

**Fig. 2:**
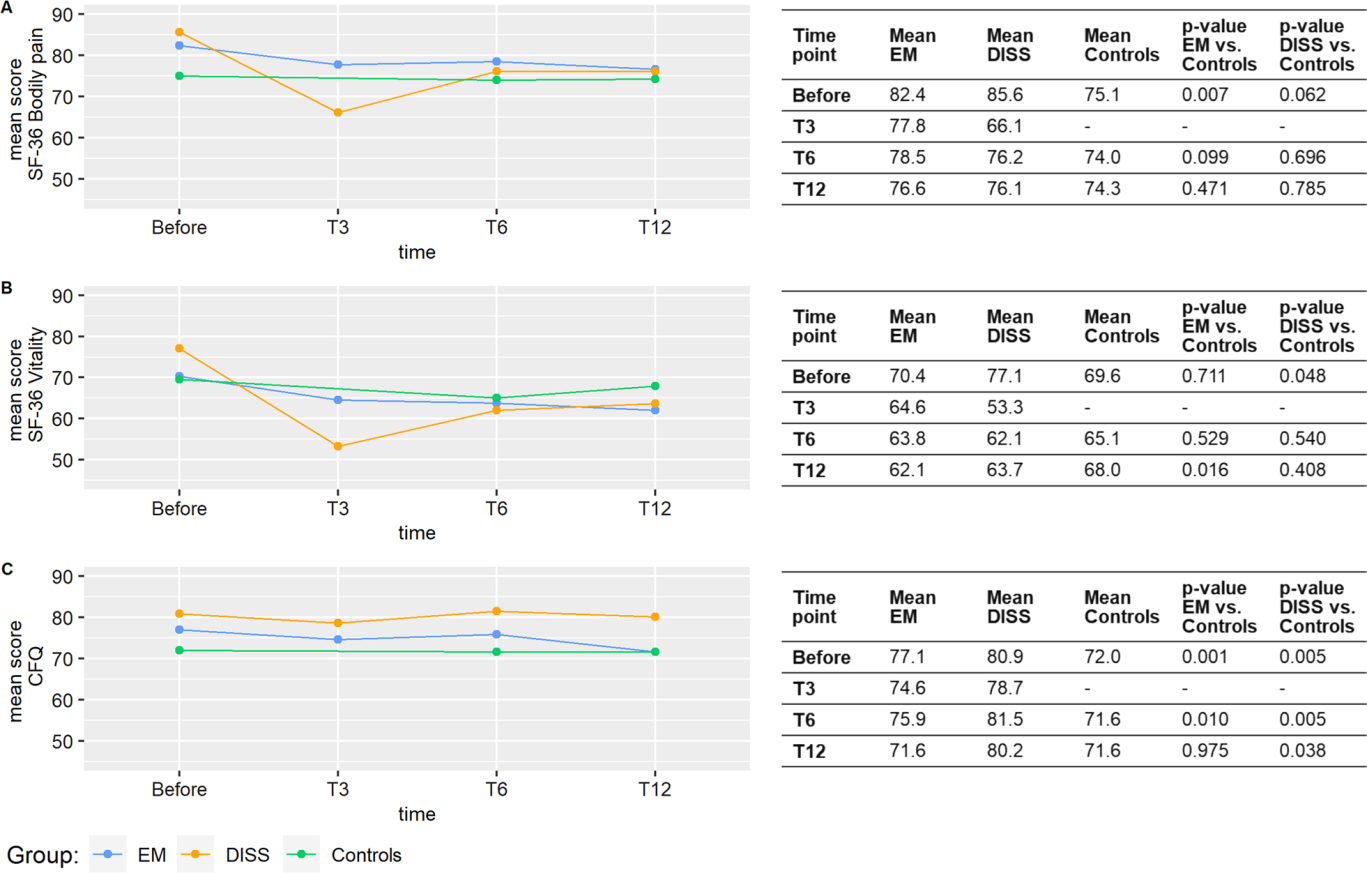
Mean scores on the standardized questionnaires by group at each time point. All scores can range from 0 to 100, a lower score represents more pain, more fatigue or more cognitive difficulties (CFQ score is inversed). Before: before LB (patient groups) or before participation (control group) retrospectively reported at inclusion. T3: 3 months after diagnosis; T6 and T12: 6 and 12 months after treatment, EM: erythema migrans, DISS: disseminated/late Lyme borreliosis

Figure 2 shows the mean scores on the standardized questionnaires and the comparison of scores between patient groups and the control group at each time point (no values for the control group at time T3). A lower score represents more pain, more fatigue or more cognitive difficulties. The SF-36 vitality score at T12 was the only score significantly worse in patients (EM) compared to controls. Disseminated/late LB patients had better vitality before LB compared to controls before participation but not anymore at follow-up, as patients’ vitality decreased. Similarly, bodily pain scores were better in LB patients before LB (significant in EM, p = 0.007) compared to controls, but the difference was smaller at follow-up (and not significant) for both patient groups. CFQ scores were significantly better in LB patients compared to controls before start and at T6. This was no longer the case for EM patients at T12, yet for disseminated/late LB, CFQ scores remained better than those of controls at all the time points. The most pronounced decline in mean score over time was seen in the disseminated/late LB group at T3 for pain and fatigue with a 23–31% lower score compared to scores before LB.

#### Any PTLDS-related symptom and impact on daily activities

Table 5 shows the proportion of participants reporting at least one of the six PTLDS-related symptoms as new or worsened, and the proportion of participants that report an additional impact on daily activities. Corresponding risk ratios are shown.

**Table 5:** Any new or worsened PTLDS-related symptom (with impact on daily activities) in patients with Lyme borreliosis compared to controls at 6 and 12 months follow-up, HUMTICK study, Belgium, 2016–2020

Time point	EM N (%)	DISS N (%)	Controls N (%)	EM vs. controls		DISS vs. controls	
				RR (95% CI)	P-value	RR (95% CI)	P-value
Total N							
6 months	120	15	128				
12 months	94	11	81				
**Any new/worse symptom**^a^^,^^b^							
6 months	24 (20.0)	7 (46.7)	13.1 (10.2)	1.96 (1.04–3.69)	**0.036**	4.58 (2.15–9.73)	**< 0.001**
12 months	21 (22.3)	7 (63.6)	9 (11.1)	2.01 (0.97–4.16)	0.060	5.73 (2.65–12.39)	**< 0.001**
**Any new/worse symptom**^a^^,^^b^ **and impact daily activities**^c^							
6 months	9 (7.5)	4 (26.7)	4.2 (3.3)	2.27 (0.72–7.19)	0.163	8.07 (2.23–29.22)	**0.002**
12 months	8 (8.5)	4 (36.4)	5 (6.2)	1.38 (0.47–4.08)	0.560	5.89 (1.83–18.98)	**0.003**

*EM* erythema migrans, *DISS* disseminated/late Lyme borreliosis, *N* number, *RR* risk ratio, *CI* confidence interval

^a^At least one of the following: muscle pain, joint pain, fatigue, memory difficulties, concentration difficulties or difficulties finding words

^b^Symptom reported as regularly present since the previous questionnaire, worsened compared to health before Lyme borreliosis and worsened score on standardized questionnaire for the symptom present (past 4 weeks)

^c^Limited on the modified GALI and having at least slight problems with daily activities on the standardized questionnaire (EQ-5D-5L, 3th question, past 4 weeks)

When an impact on daily activities was considered, the proportions decreased in all groups by 43–68% depending on the time point and group (Table 5). For this outcome, no statistically significant difference was found between EM patients and the control group at either 6- or 12-months follow-up. For the disseminated/late LB group, despite the small sample size, both RRs were significant.

### PTLDS in LB patients

Twelve EM patients were excluded from the PTLDS-analysis as they reported a history of unexplained or undiagnosed extreme fatigue, widespread musculoskeletal pain or cognitive difficulties or were previously diagnosed with fibromyalgia or chronic fatigue syndrome.

After combining new or worsened symptoms and impact on daily activities at the different time points in order to include the time frame proposed by the IDSA (Additional file 2), the estimated proportion of LB patients developing PTLDS equaled 5.9% (95% CI 2.7–12.9) in EM patients and 20.9% (95% CI 6.8–64.4) in disseminated/late LB patients (Table 6). Characteristics of PTLDS patients (non-imputed) are shown in Additional file 4. Three EM patients (2.8%) fulfilled the complete PTLDS definition at T6 as symptoms impacting daily activities were present during the first 6 months after treatment, one of which still at T12. The other three EM patients with PTLDS fulfilled the complete definition only at T12. Their symptoms started before T3 or T6, but were not yet present during 6 months or did not yet impact daily life activities at 6 months follow-up, yet did so afterwards. In the group of disseminated/late LB patients, three patients had PTLDS at T6 (20.0%) of which 2 still at T12. For none of the patients, an explanatory cause was reported for all PTLDS-related symptoms.

**Table 6:** Incidence of post-treatment Lyme disease syndrome (PTLDS) using different inclusion or outcome criteria, HUMTICK study, Belgium, 2016–2020

	EM patients		DISS patients	
	PTLDS^a^/N	Proportion (95% CI)	PTLDS^a^/N	Proportion (95% CI)
**Baseline**	6.4/108	5.9 (2.7–12.9)	3.15/15	20.9 (6.8–64.4)
1: Possible cases included	6.4/113	5.6 (2.6–12.3)	4.15/18	23.0 (9.0–58.8)
2: Insufficiently treated excluded^b^	5.4/104	5.2 (2.2–12.2)	3.15/14	22.4 (7.3–69.0)
3: No standardized questionnaires	9.45/108	8.7 (4.7–16.4)	3.25/15	21.5 (7.0–66.0)
4: No imputation	6/105	5.7 (2.6–12.4)	3/13	23.1 (8.6–62.3)

*EM* erythema migrans, *DISS* disseminated/late Lyme borreliosis, *PTLDS* post-treatment Lyme disease syndrome, *N* number, *CI* confidence interval

^a^The decimals in the numbers of PTLDS cases are the result of imputing the outcome in the censored patients, with having PTLDS in only part of the 20 repetitive imputations

^b^Assessment based on prescribed treatment

#### Risk factors for the development of PTLDS

In a univariate analysis including all LB patients, the risk of PTLDS was higher for the disseminated/late LB group compared to the EM group with a RR of 3.53 (95% CI 0.98–12.68, p = 0.053), borderline non-significant. In the EM group, univariate analyses with region, pulmonary disease and joint pain before LB, number of symptoms other than EM and having multiple EM at diagnosis showed a p-value < 0.25 (Additional file 5). After backwards exclusion from the multivariable analysis, none of the variables were significant.

#### Alternative scenarios PTLDS

Estimating the incidence of PTLDS using different inclusion criteria or without imputation provided similar results than the baseline scenario (Table 6 and Additional file 6). When a definition with higher sensitivity was used for new or worsened symptoms, by not taking into account a worsened standardized questionnaire score as a criterion, the proportion of PTLDS in EM patients increased to 8.7% (95% CI 4.7–16.4).

## Discussion

Several studies have investigated non-specific persistent symptoms after antibiotic treatment for LB, but only few were prospective cohort studies including a non-LB control group [4–6, 35–37] and few have applied the full case definition for PTLDS [16]. In this prospective study, extensive efforts were made to apply the different components of the definition to identify patients with PTLDS. In order to allow comparison with previously reported results and to show the impact of the definitions used, different steps were followed to present and discuss the study results.

First, we compared the occurrence of several new or worsened symptoms between LB patients and controls. In this separate symptom analysis, only the risk of fatigue and pain were significantly higher in LB patients compared to controls. Although multiple studies have reported high frequencies of cognitive difficulties after LB [11, 15, 38], this was not the case in our study.

Secondly, the occurrence of at least one of the symptoms listed in the IDSA PTLDS definition was compared between LB patients and controls including an assessment of the impact on daily activities. When such impact was not taken into account, the risk of either widespread musculoskeletal pain, fatigue or cognitive difficulties was significantly higher in EM patients compared to controls at 6 months follow-up (20.0% vs 10.2% respectively). Three prospective European studies that reported new or worsened symptoms without impact, yet excluding other causes, reported a somewhat lower symptom proportion of 4.6–10.7% in solitary EM patients and 15.9% in MEM patients at 6 months follow-up and 2.2–7.3% at 12 months follow-up [4–6]. On the other hand, a study in the US reported a higher proportion compared to our results, with 36% of patients reporting new-onset subjective symptoms at 6 months follow-up, yet they included EM patients with systemic symptoms only [11, 39]. Since LB is caused by different genospecies in Europe and America, a difference might be observed in the occurrence of PTLDS between European and American patients [1, 40]. When, however, impact on daily activities was considered, our study did not find a significant difference between EM patients and controls in the occurrence of any of the PTLDS-related symptoms at either 6- or 12-months follow-up, indicating a predominantly favorable evolution after treatment for EM. In general, the proportions of patients and controls that reported symptoms reduced by 43 to 68% due to the addition of the impact on daily activities as a criterion in the analysis, highlighting the importance of the definitions used. In contrast to our results, Ursinus et al. [37] did find significant differences in the prevalence of persistent symptoms between EM patients and controls in a large prospective study recently conducted in the Netherlands. Yet, they defined and evaluated persistent symptoms differently, namely as impaired scores on standardized questionnaires for fatigue, pain or cognitive impairment, during at least 6 months, with onset < 6 months. As such, they found the prevalence of these persistent symptoms to be 3.9% and 6.0% higher in EM patients compared to two different control groups respectively (general population and persons with a tick bite without LB) [37].

In the group of disseminated/late LB patients, our study did find a significantly higher risk of any PTLDS-related symptom compared to controls, both with or without considering impact on daily activities. The study by Ursinus et al. [37] also reported a significant difference between disseminated LB patients and controls, with a prevalence of persistent symptoms that was 11.0% and 13.1% higher in patients compared to the two control groups respectively. In a review of 44 studies on LNB, the estimated mean proportion of symptoms was 28% higher in patients compared to controls [13]. Other studies have also identified dissemination of LB as a risk factor for the development of persisting symptoms compared to EM [6, 41].

In general, a high background prevalence of PTLDS-related symptoms was present in our study with 51% of EM patients, 40% of disseminated/late LB patients and 67% of controls reporting at least one of the six PTLDS-related symptoms in the months before LB onset or before participation (Table 2). This prevalence differed significantly between LB patients and controls. Similarly, several mean standardized questionnaire scores were better in the patient cohorts before LB compared to controls before participation. It needs to be noted that these pre-Lyme prevalences and scores were reported retrospectively at inclusion and recall bias might have occurred. On the other hand, it is possible that LB patients are somewhat more active than the general population, as tick bites often occur during active outdoor activities. Other studies analyzing overall prevalence in patients and controls, also found proportions as high as 70–85% experiencing one of the symptoms assessed at baseline or follow-up [5, 6, 42].

In the final step of the analysis, we estimated the proportion of patients developing PTLDS in EM patients and the disseminated/late LB patients, according to the full IDSA case definition. Our study estimated the proportion of PTLDS to be 5.9% (95% CI 2.7–12.9) in EM patients and 20.9% (95% CI 6.8–64.4) in disseminated/late LB patients. Belonging to the latter group increased the risk of PTLDS with a RR of 3.53 (95% CI 0.98–12.68, p = 0.053). To our knowledge, no study has previously looked at the occurrence of new or worsened symptoms, including the impact on daily activities and the time frame as criteria for identification of PTLDS cases. Three prospective European studies mentioned above reported no cases of PTLDS at 12 months follow-up as there was no reduction in previous activity levels. Yet, it seems that the latter was not assessed at 6 months follow-up [4–6]. Two prospective studies in the US reported 5.6% and 11% PTLDS in EM patients with systemic symptoms (flu-like symptoms or dissemination EM) but they were followed-up for 6 months only [35, 39]. In our study, only three out of 108 EM patients (2.8%) fulfilled the complete PTLDS definition at 6 months follow-up, yet 5.9% did at T6 or T12, highlighting the importance of longer follow-up and combination of different follow-up points in PTLDS research to allow proper inclusion of the time frame proposed by the IDSA. It needs to be noted that newer clinical guidelines of the IDSA published in 2021 do not include a definition for PLDS or PTLDS to avoid its use in clinical settings. The definition was previously also proposed for research purposes only [25, 43].

No significant additional risk factors for PTLDS, could be identified in the study. Previous studies have reported MEM, delay in treatment, more symptoms at diagnosis, some specific symptoms at diagnosis such as fever, headache, neck stiffness but also older age, female gender, more comorbidities or fatigue in the past year, as a risk factor for post-treatment symptoms in LB patients, yet not always confirmed by other studies [6–8, 41, 44–48]. Further research with larger sample sizes is needed. To date, also the cause of PTLDS remains unknown. One of the hypotheses is that it is a post-infectious syndrome as seen with other infectious diseases such as Epstein–Barr virus infections, Q-fever and now also COVID-19 [49, 50]. More research into long-COVID that will be performed might contribute to the understanding of the pathophysiology of these post-infectious syndromes, yet it remains unknown if they share a common mechanism.

Important strengths of the current study are the prospective design, the follow-up of a control group, the use of standardized questionnaires, the assessment of health before LB or participation and the analysis of different outcomes and alternative scenarios. The latter allowed to assess, and to show, the impact of changing the case definition and study population on the results. In our study, inclusion of possible cases for the PTLDS analysis didn’t change the results a lot, yet it concerned only few additional patients (Additional file 6). Also, when only sufficiently treated patients were retained from the baseline PTLDS analysis, as proposed by the IDSA definition, the proportions of patients with PTLDS did not differ significantly compared to the baseline analysis (Additional file 6). Including all patients, however, is closer to the real-life situation where patients are still not treated optimally, as was observed in our study. Even though treatment recommendations were provided to the GPs, a high number of patients (35.6% of EM patients in the PTLDS analysis) was treated with a higher dose or longer period than advised in Belgian guidelines [51].

There are also some limitations to this study. For all groups, predefined sample sizes could not be included [22]. There were unexpected difficulties, mainly in recruiting enough patients with well documented confirmed disseminated/late LB. As a consequence the sample size of this group was small which causes confidence intervals to be large and could increase the risk of overestimating effect sizes [52]. These difficulties with inclusion can be due to strict case definitions used, in which different laboratory results need to be available. In addition, the disseminated/late LB group was heavily skewed to males (13 males vs. 2 women) and the limited sample size precluded confounders to be added to the analysis. When age and gender were added in the EM group, the same symptoms remained significant, yet any new/worse PTLDS related symptom was also significant at T12 (p = 0.04, results not shown). All results are based on patient reports only, there was no involvement of a GP or study investigator in the patient follow-up. Since the PTLDS definition comprises the exclusion of symptoms caused by another illness, participants were asked to report a cause, if known, of their symptoms. Yet, the cause might be difficult to assess and was often missing, it is therefore possible that some symptoms were related to another cause. Furthermore, there is a possible psychological effect of receiving a certain diagnosis, such as Lyme borreliosis, on the development of symptoms after treatment in the LB group, as opposed to the control group.

## Conclusions

In this study the occurrence of PTLDS was confirmed in both LB cohorts, with a higher percentage in patients with disseminated/late LB. To our knowledge, this is the first study that allowed estimating the proportion of PTLDS as defined by IDSA (2006), including strict criteria on symptoms, impact on daily activities and the time frame. Additional insight was gained from intermediate analysis steps. Of all non-specific symptoms, a higher risk of pain and fatigue was shown in LB cohorts compared to controls. Since the proportion of patients with non-specific symptoms after LB changed substantially when considering the impact on daily activities and the time criterion, future research should include these different components. Yet, development and validation of standardized methods that are easily applicable to assess these components remains important. As non-specific symptoms are highly prevalent in the general population and occur without being disabling, inclusion of a non-LB control group is also essential in these future studies. In order to have a sufficiently large sample size, allowing identification of risk factors and possible causes for PTLDS, international collaborations should be encouraged.

## Supplementary Information

**Additional file 1: Table S1.** Case definitions for inclusion in the HUMTICK study, Belgium, 2016–2020 [1].

**Additional file 2: Table S2.** Definition post-treatment Lyme disease syndrome according to the Infectious Diseases Society of America (IDSA) and HUMTICK project.

**Additional file 3: Figure S1.** Flowchart patient inclusion.

**Additional file 4: Table S3.** Characteristics of patients with PTLDS (not-imputed only), HUMTICK study, Belgium, 2016–2020.

**Additional file 5: Table S4.** Associations of possible risk factors with the development of post-treatment Lyme disease syndrome, HUMTICK study, Belgium, 2016–2020—univariate log-binomial analysis.

**Additional file 6.** Alternative scenarios to estimate the proportion of PTLDS.

## Data Availability

The datasets analyzed during the current study are available from the corresponding author on reasonable request.

## Abbreviations

LB: Lyme borreliosis
*B. **burgdorferi* s.l.: *Borrelia burgdorferi* sensu lato
EM: Erythema migrans
MEM: Multiple erythema migrans
LNB: Lyme neuroborreliosis
LA: Lyme arthritis
ACA: Acrodermatitis chronica atrophicans
PLDS: Post-Lyme disease syndrome
PTLDS: Post-treatment Lyme disease syndrome
IDSA: Infectious Disease Society of America
T0: Inclusion, T1/T3: 1/3 months after inclusion
T6/T12/T24: 6/12/24 months after treatment completion
SF-36 BP: SF-36 bodily pain
SF-36 VT: SF-36 vitality
CFQ: Cognitive Failure Questionnaire
GALI: Global activity limitation indicator
CI: Confidence interval
LTFU: Loss to follow-up
