# Supplementary material for: Non-specific symptoms and post-treatment Lyme disease syndrome in patients with Lyme borreliosis: a prospective cohort study in Belgium (2016–2020)
Source: BMC Infect Dis. 2022 Sep 28;22:756. doi: 10.1186/s12879-022-07686-8 (PMC9518937; doi:10.1186/s12879-022-07686-8)
Supplement: Supplementary file 1 — Additional file 1: Table S1. Case definitions for inclusion in the HUMTICK study, Belgium, 2016–2020 [1]. [file 12879_2022_7686_MOESM1_ESM.docx]

# Additional file 1

**Table S1. Case definitions for inclusion in the HUMTICK study, Belgium, 2016-2020** [1]

|  | **Case definition** |
| --- | --- |
| **Cohort 1: EM** | - Clinical presentation of EM or MEM, confirmed by a GP |
| **Cohort 2: Confirmed disseminated Lyme borreliosis** | - Positive polymerase chain reaction (PCR) or culture   OR   - Positive serology AND at least one clinical manifestation compatible with disseminated/late Lyme borreliosis (confirmed by the treating physician) [2].   [see below for the specific description of this case definition] |

**Specific description of the case definitions for (confirmed) cases of disseminated Lyme borreliosis which will be included in the HUMTICK study:**

**Adapted from CDC and EUCALB case definitions*** [2, 3]

**Laboratory evidence:**

- Isolation or positive PCR from tissue or body fluid

OR

- Positive serology (using the two-tier ELISA and Western Blot)

OR

- Pleocytosis and antibody production against *B. burgdorferi* in the cerebrospinal fluid (CSF), evidenced by a higher titer of antibody in CSF than in serum (positive antibody index) (always necessary for Lyme neuroborreliosis) [4]

**AND clinical evidence:**

at least one of the following clinical manifestations corresponding with disseminated Lyme borreliosis, when an alternate explanation is not found:

- **Skin manifestation:**

Multiple erythema migrans (included in cohort 1 in the current study) or acrodermatitis chronica atrophicans.

- **Musculoskeletal system (Lyme arthritis):**

Recurrent episodes (weeks or months) of objective joint swelling in one (commonly the knee) or a few joints, sometimes followed by chronic arthritis in one or a few joints. The following manifestations are not considered for inclusion: chronic progressive arthritis not preceded by brief attacks, chronic symmetrical polyarthritis, and arthralgia, myalgia, or fibromyalgia syndromes alone.

- **Nervous system (neuroborreliosis):**

Any of the following neurological symptoms (alone or in combination): lymphocytic meningitis; cranial neuritis, particularly facial palsy (uni- or bilateral); radiculitic pain; or, rarely encephalomyelitis. Headache, fatigue, paresthesia, or mildly stiff neck alone, are not considered for inclusion.

- **Cardiovascular system (carditis):**

Acute onset of high-grade (2nd-degree or 3rd-degree) atrioventricular conduction defects that resolve in days to weeks and are sometimes associated with myocarditis. Palpitations, bradycardia, bundle branch block, or myocarditis alone are not considered for inclusion.

* Belgium is an area endemic for Lyme Borreliosis, therefore all included patients fulfill the criterion of exposure to *B. burgdorferi.*

# References

1. Geebelen L, Lernout T, Kabamba-Mukadi B, Saegeman V, Sprong H, Van Gucht S, et al. The HUMTICK study: protocol for a prospective cohort study on post-treatment Lyme disease syndrome and the disease and cost burden of Lyme borreliosis in Belgium. Archives of Public Health. 2017;75:42.

2. Stanek G, Fingerle V, Hunfeld K-P, Jaulhac B, Kaiser R, Krause A, et al. Lyme borreliosis: Clinical case definitions for diagnosis and management in Europe. Clinical Microbiology and Infection. 2011;17:69–79.

3. Centers for Disease Control and Prevention. Lyme Disease (Borrelia burgdorferi) 2011 Case Definition. https://wwwn.cdc.gov/nndss/conditions/lyme-disease/case-definition/2011/.

4. Commission implementing decision (EU) 2018/945 of 22 June 2018 on the communicable diseases and related special health issues to be covered by epidemiological surveillance as well as relevant case definitions. 2018. https://eur-lex.europa.eu/legal-content/EN/TXT/PDF/?uri=CELEX:32018D0945&from=EN#page=29. Accessed 21 May 2021.
