# Supplementary material for: Non-specific symptoms and post-treatment Lyme disease syndrome in patients with Lyme borreliosis: a prospective cohort study in Belgium (2016–2020)
Source: BMC Infect Dis. 2022 Sep 28;22:756. doi: 10.1186/s12879-022-07686-8 (PMC9518937; doi:10.1186/s12879-022-07686-8)
Supplement: Supplementary file 2 — Additional file 2: Table S2. Definition post-treatment Lyme disease syndrome according to the Infectious Diseases Society of America (IDSA) and HUMTICK project. [file 12879_2022_7686_MOESM2_ESM.docx]

# Additional file 2

**Table S2.** Definition post-treatment Lyme disease syndrome according to the Infectious Diseases Society of America (IDSA) and HUMTICK project.

| **Theoretical definition PTLDS** | |
| --- | --- |
| **IDSA (2006)** | **HUMTICK** |
| ‘Documented episode of early or late Lyme disease, case definition of the Centers for Disease Control and Prevention’ | ‘Confirmed Lyme disease based on case definitions’ (Additional file 1) |
| ‘Treatment with generally accepted regimen with resolution/stabilization of objective manifestations’ | ‘Treatment prescribed by participating GP or specialist’ |
| **Subjective PTLDS symptoms:** | |
| ‘Onset of any of the following:  - fatigue,  - widespread musculoskeletal pain,  - complaints of cognitive difficulties’ | ‘Onset or worsening of any of the following 6 symptoms: fatigue (1); widespread musculoskeletal pain expressed as: muscle pain (2), joint pain (3) at more than one place of the body; cognitive difficulties expressed as: memory problems (5), difficulties concentrating (6) or problems finding words (7)’ |
| ‘Symptom onset within 6 months of the diagnosis of Lyme disease and persistence of continuous or relapsing symptoms for at least a 6 month period after completion of antibiotic therapy’ | ‘Symptom onset within 6 months of the diagnosis of Lyme disease and regularly present for at least 6 months after antibiotic therapy’  = Onset before T3 and regularly present up and until T6 (and possibly also between T6 and T12)  OR Onset before T6 and regularly present between T6 and T12 |
| **Impact daily activities:** | |
| ‘Subjective symptoms are of such severity that, when present, they result in substantial reduction in previous levels of occupational, educational, social, or personal activities’ | ‘Subjective symptoms are of such severity that they impact daily activities (e.g. work, school, hobbies or other). Start of impact did not necessarily had to coincide with start symptoms, it could start later but needed to last 6 months’ |
| **Exclusion:** | |
| A diagnosis of an underlying disease or condition that might explain the patient’s symptoms | If a patient reports another cause, which explains the new occurrence or worsening of a symptom (e.g. acute disease, newly diagnosed disease, flare-up of existing disease,..) the symptom is not included as new or worsened symptom for the final PTLDS proportion |
| A diagnosis of fibromyalgia or chronic fatigue syndrome before the onset of Lyme disease | Previously diagnosed fibromyalgia, chronic fatigue syndrome or chronic Lyme disease |
| A prolonged history of undiagnosed or unexplained somatic complaints, such as musculoskeletal pains or fatigue, before the onset of Lyme disease | A history of unexplained or undiagnosed complaints such as extreme fatigue, widespread musculoskeletal pain or cognitive difficulties |
| An active, untreated, well-documented coinfection such as babesiosis | EM patients were tested for other tick-borne pathogens at diagnosis, yet only with PCR. None were positive (personal communication) |
| The presence of objective abnormalities on physical examination or on neuropsychologic testing that may explain the patient’s complaints | No additional examination or testing carried out at follow-up in the scope of this study |
| Laboratory or imaging abnormalities that might suggest an undiagnosed process distinct from post–Lyme disease syndrome | No additional examination or testing carried out at follow-up in the scope of this study |
| Although testing by either culture or PCR for evidence of *Borrelia burgdorferi* infection is not required, should such testing be done by reliable methods, a positive result would be an exclusion | No additional examination or testing carried out at follow-up in the scope of this study |
