# Supplementary material for: Non-specific symptoms and post-treatment Lyme disease syndrome in patients with Lyme borreliosis: a prospective cohort study in Belgium (2016–2020)
Source: BMC Infect Dis. 2022 Sep 28;22:756. doi: 10.1186/s12879-022-07686-8 (PMC9518937; doi:10.1186/s12879-022-07686-8)
Supplement: Supplementary file 3 — Additional file 3: Figure S1. Flowchart patient inclusion. [file 12879_2022_7686_MOESM3_ESM.docx]

# Additional file 3


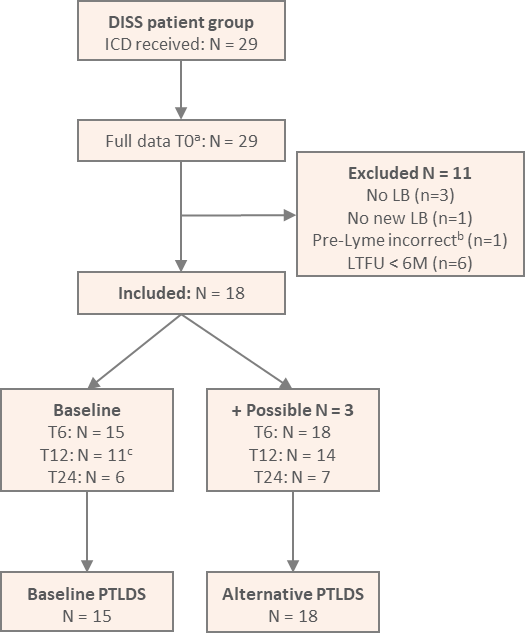

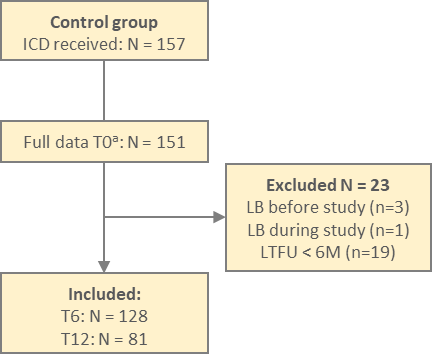

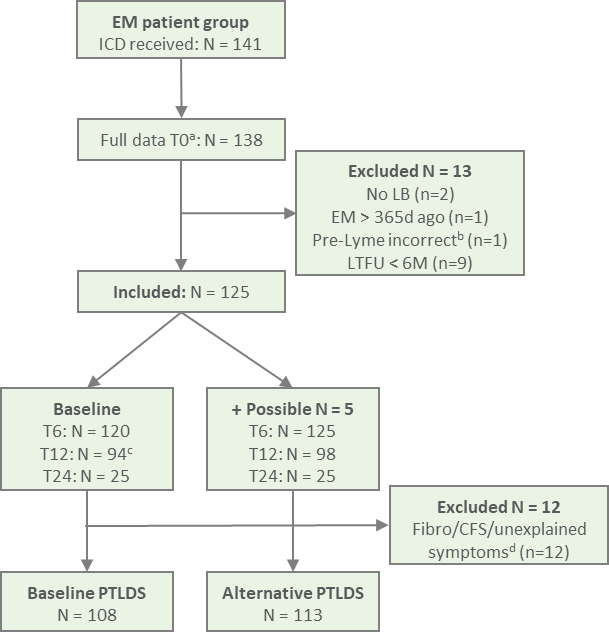
**Figure S1:** flowchart patient inclusion

EM: erythema migrans, ICD: Informed Consent Document, DISS: disseminated/late Lyme borreliosis, LB: Lyme borreliosis, T0: Inclusion, LTFU: Loss to follow-up, T6: 6 months after treatment, T12: 12 months after treatment , Fibro: fibromyalgia, CFS: Chronic Fatigue Syndrome

^a^ Exclusion only if complete or complete part of questionnaire T0 was missing (e.g. GP part, patient part or standardized questionnaire part)

^b^ One EM patient reported chronic LB as a comorbidity due to a LB infection years ago and assessed pre-Lyme health erroneously as his/her health before that LB diagnosis. One disseminated LB patient assessed his health before LB as his health before a rheumatoid arthritis diagnosis causing similar symptoms

^c^ Of the 125 EM patients at T6, 13 did not respond at T12 (true LTFU) and 14 were not followed-up as they started to participate late in the study. For disseminated/late LB, all patients responded at T12, 4 patients were not followed-up as they were included late. For the control group, 14 did not respond, whereas 32 were not followed-up

^d^ History of unexplained or undiagnosed extreme fatigue, widespread musculoskeletal pain or cognitive difficulties
