# Supplementary material for: Non-specific symptoms and post-treatment Lyme disease syndrome in patients with Lyme borreliosis: a prospective cohort study in Belgium (2016–2020)
Source: BMC Infect Dis. 2022 Sep 28;22:756. doi: 10.1186/s12879-022-07686-8 (PMC9518937; doi:10.1186/s12879-022-07686-8)
Supplement: Supplementary file 4 — Additional file 4: Table S3. Characteristics of patients with PTLDS (not-imputed only), HUMTICK study, Belgium, 2016–2020. [file 12879_2022_7686_MOESM4_ESM.docx]

**Additional file 4**

| PTLDS  case | Age | Gender | T0 | PTLDS^1^ | New/worse PTLDS related symptoms² | Symptom start | Symptom duration | Impact start | Impact duration | Other symptoms at follow-up |
| --- | --- | --- | --- | --- | --- | --- | --- | --- | --- | --- |
| #1 | 50-54 | F | EM | **≤T1-24**  (end FU) | Muscle pain, joint pain (T3)  Fatigue (T3, T12)  Memory (T3, T6, T12, T24)  Wording (T3, T12, T24)  Concentration (T12) | ≤T1 | 24m  (end FU) | ≤T1 | 24m  (end FU) | Excessive sleeping (T12) |
| #2 | 55-59 | F | EM | **≤T1-6** | Fatigue (T3, T6, T12)  Muscle pain, joint pain (T12) | ≤T1 | 12m  (end FU) | ≤T1 | 6m | Headache (T3), Night sweats (T3),  difficulties falling asleep (T12) |
| #3 | 55-59 | F | EM | **≤T1-6** | Muscle pain (T3,T6)  Joint pain (T3=NA, T6)  Severity fatigue = NA (T3, T6) | ≤T1 | 6m | ≤T1 | 6m | Sensory disorders (T6),  Night sweats (T6, T3 severity=NA)  Excessive sleeping (T6, T3 severity=NA) |
| #4 | 60-64 | M | EM | **T6-12** | Muscle pain (T6, T12)  Joint pain (T3, T12)  Memory (T6), wording (T6) | ≤T1 | 12m | T6 | 6m | Headache (T3),  difficulties falling asleep (T3, T12), |
| #5 | 50-54 | M | MEM | **T6-12** | Fatigue (T6, T12)  Memory, wording, concentration (T12) | T3 | 21m  (end FU) | T6 | 6m | Excessive sleeping (T6, T12),  Sensory disorders T12=NA,  difficulties falling asleep T12=NA |
| #6 | 50-54 | M | EM | **T6-12** | Fatigue (T6, T12)  Memory (T6) | T3 | 9m | T6 | 6m | Headache (T6), sensory disorders (T6) |
| #7 | 50-54 | M | Early LNB | **≤T1-24** (end FU) | Fatigue (T3, T6, T12, T24)  Joint pain (T3, T24) | ≤T1 | 24m  (end FU) | ≤T1 | 24m  (end FU) | / |
| #8 | 45-49 | M | LA | **≤T1-T12** (end FU) | Fatigue (T3, T6, T12)  Muscle pain (T6)  Joint pain (T12) | ≤T1 | 12m  (end FU) | ≤T1 | 12m  (end FU) | Initial swollen knee, until T6,  initial joint pain knee (T3, T6, T12) |
| #9 | 25-29 | M | Early LNB | **≤T1-6** | Fatigue (T3, T6, T12) | ≤T1 | 12m  (end FU) | ≤T1 | 6m | Neurological pain multiple locations (T3), one location (T6, T12) |

**Table S3.** Characteristics of patients with PTLDS (not-imputed only), HUMTICK study, Belgium, 2016-2020

PTLDS: post-treatment Lyme disease syndrome, F: female, M : male, EM: erythema migrans, MEM: multiple erythema migrans, LNB: Lyme neuroborreliosis, LA: Lyme arthritis, T0: diagnosis, T3: 3 months after diagnosis; T6, T12 and T24: 6, 12 and 24 months after treatment, NA: missing, FU: follow-up, m: months,

^1^ Period for which both the symptom and impact criterion are fulfilled, both based on general question and standardized questionnaire/question

² Muscle pain or joint pain at more than 1 location, fatigue, memory problems (forgetfulness), difficulties concentrating or problems finding words, without other cause reported
