# Supplementary material for: Non-specific symptoms and post-treatment Lyme disease syndrome in patients with Lyme borreliosis: a prospective cohort study in Belgium (2016–2020)
Source: BMC Infect Dis. 2022 Sep 28;22:756. doi: 10.1186/s12879-022-07686-8 (PMC9518937; doi:10.1186/s12879-022-07686-8)
Supplement: Supplementary file 5 — Additional file 5: Table S4. Associations of possible risk factors with the development of post-treatment Lyme disease syndrome, HUMTICK study, Belgium, 2016–2020—univariate log-binomial analysis. [file 12879_2022_7686_MOESM5_ESM.docx]

# Additional file 5

**Table S4.** Associations of possible risk factors with the development of post-treatment Lyme disease syndrome, HUMTICK study, Belgium, 2016-2020 - Univariate log-binomial analysis

|  | | **Category** | **N^1^** | **PTLDS   N (%)** | **RR (95% CI)** | **P-value** |
| --- | --- | --- | --- | --- | --- | --- |
| **Total** | | | **108** | **6.4 (5.9)** |  | |
| **Gender** | | | | | |  |
|  | Male | | 45 | 3.0 (6.7) | Reference | **-** |
|  | Female | | 63 | 3.4 (5.4) | 0.80 (0.17-3.81) | 0.779 |
| **Age** | | | | | |  |
|  | Years | |  | | 1.01 (0.96-1.06) | 0.681 |
| **Region^2^** | | | | | | 0.183** |
|  | Flanders | | 77 | 2.4 (3.1) | Reference | **-** |
|  | Wallonia | | 29 | 4.0 (13.8) | 4.52 (0.88-23.20) | 0.071 |
|  | Brussels | | 2 | 0.0 (0.0) | 0.00 (0.00-Inf) | 0.996 |
| **Highest completed education** | | | | | |  |
|  | Lower | | 46 | 3.4 (7.4) | Reference | **-** |
|  | Higher | | 62 | 3.0 (4.8) | 0.66 (0.14-3.14) | 0.600 |
| **Work** | | | | | | 0.889 |
|  | Full-time | | 44 | 2.4 (5.5) | Reference | **-** |
|  | Part-time | | 24 | 3.0 (12.5) | 2.34 (0.42-12.99) | 0.328 |
|  | Student | | 4 | 0.0 (0.0) | 0.00 (0.00-Inf) | 0.998 |
|  | Retired | | 28 | 0.0 (0.0) | 0.00 (0.00-Inf) | 0.995 |
|  | Not working, other | | 8 | 1.0 (12.5) | 2.34 (0.24-23.05) | 0.463 |
| **Inclusion period** | | | | | |  |
|  | Spring/Summer | | 88 | 5.4 (6.1) | Reference | **-** |
|  | Autumn/Winter | | 20 | 1.0 (5.0) | 0.82 (0.10-6.76) | 0.851 |
| **Comorbidity** | | | | | |  |
|  | Musculoskeletal disease | | 15 | 1.0 (6.7) | 1.15 (0.14-9.39) | 0.893 |
|  | Heart disease | | 8.1 | 0.0 (0.0) | 0.00 (0.00-Inf) | 0.994 |
|  | Pulmonary disease | | 8 | 1.4 (17.5) | 3.30 (0.45-23.92) | 0.234** |
|  | Thyroid disorder | | 6 | 0.0 (0.0) | 0.00 (0.00-Inf) | 0.995 |
|  | Other possibly impacting disease^3^ | | 9 | 1.0 (11.1) | 2.05 (0.26-15.98) | 0.492 |
|  | Other non-impacting disease^4^ | | 26 | 0.0 (0.0) | 0.00 (0.00-Inf) | 0.993 |
| **PTLDS-related symptoms in the months before Lyme borreliosis** | | | | | |  |
|  | Muscle pain | | 22.4 | 2.4 (10.7) | 2.24 (0.44-11.39) | 0.327 |
|  | Joint pain | | 27.6 | 3.4 (12.3) | 3.28 (0.70-15.42) | 0.132** |
|  | Fatigue | | 28 | 1.4 (5.0) | 0.75 (0.10-5.95) | 0.786 |
|  | Memory difficulties | | 11.2 | 0.0 (0.0) | 0.00 (0.00-Inf) | 0.993 |
|  | Concentration difficulties | | 11.3 | 0.1 (1.3) | 0.00 (0.00-Inf) | 0.994 |
|  | Wording difficulties | | 15.9 | 0.2 (1.3) | 0.00 (0.00-Inf) | 0.995 |
| **Symptoms at diagnosis** | | | | | |  |
|  | N symptoms other than EM^5^ | |  | | 1.17 (0.90-1.52) | 0.242** |
|  | Multiple EM^6^ | | 5 | 1.0 (20.0) | 3.83 (0.53-27.44) | 0.179** |
|  | Duration EM | |  | | 1.01 (0.97-1.05) | 0.547 |
|  | EM diameter^7^ | |  | | 1.05 (0.94-1.16) | 0.379 |
| **Antibiotic treatment^8^** | | | | | | 0.266 |
|  | Recommended | | 65.5 | 3.0 (4.6) | Reference | **-** |
|  | > Recommended | | 38.5 | 2.4 (6.2) | 1.34 (0.23-7.62) | 0.741 |
|  | < Recommended | | 4 | 1.0 (25.0) | 5.46 (0.70-42.42) | 0.103 |

** p-value < 0.25, included in multivariable model

N: number, PTLDS: post-treatment Lyme disease syndrome, RR: risk ratio, CI: confidence interval, LB: Lyme borreliosis

^1^ The decimal numbers are the result of imputing the variables when missing, with the same value only in part of the repetitive imputations.

^2^ One patient living in the Netherlands and one in France, close to the Belgian border, were diagnosed by a GP in Belgium and included in the region Flanders and Wallonia respectively.

^3^ Other diagnosis with impact expected on fatigue, widespread pain or cognitive difficulties: sleep apnea, anemia, cancer within past 2 years, hemochromatosis, Ehler-Danlos, depression, Attention Deficit Disorder, hepatitis, Crohn's disease, chronic pelvic pain.

^4^ Other diagnosis with no or limited impact expected on fatigue, widespread pain or cognitive difficulties: including gastro-esophageal or intestinal diseases, urinary, eye, skin, liver or gynecological diseases.

^5^ Number of symptoms from list: fever, headache, nausea, muscle pain, joint pain, swollen joints, neck pain, night sweats, fatigue, memory problems, difficulties concentrating and problems finding words

^6^ Multiple erythema’s reported, not necessarily confirmed with serology.

^7^ When EM diameter was <5cm (n=10.6), the exact diameter was by default missing and a random value between 2 and 4 cm was imputed for the statistical analysis. When no circle, the average of the longest and shortest diameter was used.

^8^ Recommended: 1st (10 days 2x100mg doxycycline), 2nd (14 days 3x500mg amoxicilline) or 3th choice (14 days 2x500mg cefuroxime) following Belgian guidelines [1] or 10 days 1x200mg doxycycline; > Recommended: longer treatment or higher dose; < Recommended: lower dose or shorter treatment. Assessment was based on prescribed treatment.

1. Belgian Antibiotic Policy Coordination Committee (BAPCOC). Recommandations: borréliose de Lyme 2017. 2017.
