# Supplementary material for: Non-specific symptoms and post-treatment Lyme disease syndrome in patients with Lyme borreliosis: a prospective cohort study in Belgium (2016–2020)
Source: BMC Infect Dis. 2022 Sep 28;22:756. doi: 10.1186/s12879-022-07686-8 (PMC9518937; doi:10.1186/s12879-022-07686-8)
Supplement: Supplementary file 6 — Additional file 6. Alternative scenarios to estimate the proportion of PTLDS. [file 12879_2022_7686_MOESM6_ESM.docx]

**Additional file 6**

**Alternative scenarios to estimate the proportion of PTLDS**

In a first alternative scenario, 5 possible EM cases and 3 possible disseminated/late LB patients were included in addition to the cases in the baseline scenario. A similar proportion of PTLDS was found in EM patients and a small increase in disseminated/late LB patients (Table 6). In a second scenario, insufficiently treated patients, more specifically 4 EM patients who received 100 mg doxycycline a day and one early LNB patient treated during 10 instead of 14 days, were excluded from the baseline scenario. The proportion of PTLDS reduced to 5.2% (95% CI 2.2-12.2) for EM and increased to 22.4% (95% CI 7.3-69.0) for disseminated/late LB (Table 6). In a third alternative scenario using a more sensitive case definition, in which standardized questionnaire scores (covering past 4 weeks) were not included in the criteria for both new or worsened symptoms and daily activities, three additional EM patients fulfilled the PTLDS definition, leading to a proportion of 8.7% (95% CI 4.7-16.4). In two out of these additional EM patients, not having an impact on the EQ-5D-5L question in the past 4 weeks, caused the patient not to fulfill the baseline definition, the third patient also did not have a worsened score on the symptom standardized questionnaires at the correct time points. No additional cases were identified in the disseminated/late LB group and there was only a small difference due to imputation. Finally, in the baseline scenario without multiple imputation, so excluding 3 EM patients and 2 disseminated/late LB patients from the analysis for which the final PTLDS status was missing (censored), the proportions equaled 5.7% (2.6-12.4) in EM patients and 23.1% (8.6-62.3) in disseminated/late LB patients.
